# ProstaMine: a bioinformatics tool for identifying subtype-specific co-alterations associated with aggressiveness in prostate cancer

**DOI:** 10.3389/fphar.2024.1360352

**Published:** 2024-05-01

**Authors:** Michael V. Orman, Varsha Sreekanth, Teemu D. Laajala, Scott D. Cramer, James C. Costello

**Affiliations:** ^1^ Department of Pharmacology, University of Colorado Anschutz Medical Campus, Aurora, CO, United States; ^2^ Department of Mathematics and Statistics, University of Turku, Turku, Finland; ^3^ University of Colorado Cancer Center, University of Colorado Anschutz Medical Campus, Aurora, CO, United States

**Keywords:** prostate cancer, molecular subtypes, NKX3-1, RB1, data mining algorithm, bioinformatics analysis

## Abstract

**Background:**

Prostate cancer is a leading cause of cancer-related deaths among men, marked by heterogeneous clinical and molecular characteristics. The complexity of the molecular landscape necessitates tools for identifying multi-gene co-alteration patterns that are associated with aggressive disease. The identification of such gene sets will allow for deeper characterization of the processes underlying prostate cancer progression and potentially lead to novel strategies for treatment.

**Methods:**

We developed ProstaMine to systematically identify co-alterations associated with aggressiveness in prostate cancer molecular subtypes defined by high-fidelity alterations in primary prostate cancer. ProstaMine integrates genomic, transcriptomic, and clinical data from five primary and one metastatic prostate cancer cohorts to prioritize co-alterations enriched in metastatic disease and associated with disease progression.

**Results:**

Integrated analysis of primary tumors defined a set of 17 prostate cancer alterations associated with aggressive characteristics. We applied ProstaMine to *NKX3-1-*loss and *RB1-*loss tumors and identified subtype-specific co-alterations associated with metastasis and biochemical relapse in these molecular subtypes. In *NKX3-1*-loss prostate cancer, ProstaMine identified novel subtype-specific co-alterations known to regulate prostate cancer signaling pathways including MAPK, NF-kB, p53, PI3K, and Sonic hedgehog. In *RB1*-loss prostate cancer, ProstaMine identified novel subtype-specific co-alterations involved in p53, STAT6, and MHC class I antigen presentation. Co-alterations impacting autophagy were noted in both molecular subtypes.

**Conclusion:**

ProstaMine is a method to systematically identify novel subtype-specific co-alterations associated with aggressive characteristics in prostate cancer. The results from ProstaMine provide insights into potential subtype-specific mechanisms of prostate cancer progression which can be formed into testable experimental hypotheses. ProstaMine is publicly available at: https://bioinformatics.cuanschutz.edu/prostamine.

## 1 Introduction

Prostate cancer (PCa) is the second most common cancer in men and the fourth most common cancer overall. In terms of mortality, PCa accounts for the fifth-most deadly cancer in men worldwide (Sung et al., 2021). Prostate cancer is a highly heterogeneous disease in both its clinical presentation and molecular features (Boyd et al., 2012). This heterogeneity makes it difficult to anticipate outcomes of newly diagnosed patients, where the vast majority of patients with localized disease will have little impact on overall survival; however, an unknown subset of 10%–15% of patients will progress with aggressive disease. The genomic landscape of PCa has been well-defined, with alterations, such as the *TMPRSS2:ERG* fusion (Tomlins et al., 2005; Taylor et al., 2010); *SPOP* mutations (Barbieri et al., 2012; Abeshouse et al., 2015); and the losses of *NKX3-1* (Emmert-Buck et al., 1995; Abdulkadir et al., 2002), *CHD1* (Augello et al., 2019), *MAP3K7* (Wu et al., 2012), and *PTEN* (Cairns et al., 1997; Wang et al., 2003), playing a role in disease development but none sufficient to drive PCa in men to become metastatic. With the exception of AR amplifications in castration-resistant and metastatic tumors (Abeshouse et al., 2015; Abida et al., 2019), few alterations are enriched or consistently associated with metastatic disease alone.

Recent efforts have focused on understanding how multiple alterations interact to drive aggressive PCa. In genetically engineered mice, co-loss of *RB1* and *PTEN* facilitates lineage plasticity and metastasis, and additional deletion of *TP53* promotes therapeutic resistance (Ku et al., 2017; Mu et al., 2017). In tumor xenograft studies, a combined alteration of *RB1* and *TP53* drives increased tumor growth, stem-like features, and therapeutic resistance to multiple antiandrogens (Nyquist et al., 2020). Co-occurring deletion of *MAP3K7* and *CHD1* is another example of coordinating interactions; this dual loss drives aggressive phenotypes both *in vitro* and *in vivo*, contributes to increased ARv7 expression, and is highly enriched in brain metastases (Rodrigues et al., 2015; Ormond et al., 2019; Jillson et al., 2021). These results indicate that aggressiveness in PCa is driven by the combination of multiple genomic loci, predominantly the loss of multiple tumor suppressors. These findings also suggest that the specific combination of alterations is important for disease development. There is a need to identify the genetic interactions that contribute to the progression of primary prostate cancer into metastatic disease in a molecular subtype-specific manner.

To systematically address this need, we developed a tool, ProstaMine, that integrates molecular and clinical data from multiple, independent PCa cohorts to identify co-alterations associated with molecular subtypes defined in primary disease that are enriched in metastasis and promote biochemical relapse. We leverage five primary PCa cohorts to identify putative molecular features, which we then use to find enriched co-alterations in a cohort of metastatic disease. We demonstrate our approach in PCa defined by the loss of *NKX3-1* or *RB1* and make ProstaMine publicly accessible to evaluate user-defined subtypes through a user-friendly R Shiny application (https://bioinformatics.cuanschutz.edu/prostamine).

## 2 Materials and methods

### 2.1 Data sources and processing

Genomic, transcriptomic, and clinical data analyzed in this study were from primary and metastatic tumors profiled in the literature (Taylor et al., 2010; Barbieri et al., 2012; Baca et al., 2013; Hieronymus et al., 2014; Abeshouse et al., 2015; Abida et al., 2019). We selected these studies based on the availability of copy number alteration (CNA), gene expression, and clinicopathologic data (Supplementary Figure S1). The *curatedPCaData* R package (v.0.99.4), which provides harmonized data and common, updated gene annotation across 19 independent PCa cohorts, was used to access all genomic, transcriptomic, and clinicopathologic data. *CuratedPCaData* allowed us to perform consistent and robust downstream analysis with details on methods and curation available in Laajala et al. (2023). Copy number data comprise discretized GISTIC2 calls (Mermel et al., 2011). Gene expression data are normalized counts transformed into z-scores relative to other tumor samples from the same study. Tumor grade data are based on the Gleason scoring system, and tumor stage data are based on the TNM staging system.

### 2.2 Alteration landscape and alteration hotspots

Copy number alteration gains (Gains) were defined as genes with a GISTIC value >0. Copy number alteration losses (Losses) were defined as genes with a GISTIC value <0 and/or genes with single-base substitutions having a predicted damaging effect on protein function, as computed by SIFT or PolyPhen-2 (Ng and Henikoff, 2003; Adzhubei et al., 2010). Gene alteration frequency was computed as the ratio of tumors with the alteration of the gene to the total number of tumors. The ratio of tumors covered was determined for each set of genes captured by alteration frequency cut-offs between 0 and 1. At each cut-off, coverage was calculated by the number of tumors with the alteration of at least one gene in the set to the total number of tumors. Alteration hotspots were defined as the contiguous loci containing five or more genes above a 10% alteration frequency cut-off. Visualization of the alteration landscape and alteration hotspots was done using the *karyoploteR* R package (v.1.22.0).

### 2.3 Alteration heatmap

A total of 17 alteration hotspot regions were selected based on the criteria from Section 2.2. Through manual inspection, we noted the alteration hotspots harbored genes with known involvement in prostate cancer or cancers of other tissues and that these genes were located either directly at or near the peak of the alteration hotspot. We selected a single cancer-associated gene to represent each of the PCa17 hotspots and then consensus clustering spanning k = 2 to k = 6 to identify the four subgroups (A-high, CG-1, CG-2, and A-low; Supplementary Figure S2)*.* Between k = 4 and k = 5, we noted a marginal change in the area under the CDF curve. Cluster memberships at k = 4 indicated four stable clusters each holding a robust number of patients (Supplementary Figure S2). We selected k = 4 as the distinct number of molecularly defined primary tumor subgroups. Clustering analysis was performed using the *ConsensusClusterPlus* R package (v.1.64.0), and the alteration heatmap was generated using the *ComplexHeatmap* R package (v.2.12.0).

### 2.4 Mutual exclusivity

Mutual exclusivity was calculated using Fisher’s exact test for all pairwise combinations of *CHD1, MAP3K7, LRP1B, ERG, SHQ1, TP53, HDAC5,* and *PTEN* alterations. The *ComplexHeatmap* R package (v.2.12.0) was used to visualize mutual exclusivity analysis across all primary tumors.

### 2.5 Survival analysis

The Kaplan–Meier analysis was performed using progression-free survival data from TCGA and Taylor studies (Taylor et al., 2010; Abeshouse et al., 2015), as reported in the *curatedPCaData* R package. The *survival* R package (v.3.5–7) was used to fit Cox proportional hazards models and compute statistics for progression-free survival times between groups using the logrank test. The *survminer* R package (v0.4.9) was used to visualize survival curves.

### 2.6 ProstaMine algorithm

ProstaMine first subsets the alteration data into two groups: patient tumors that are wild-type, or diploid, for the selected alteration (WT tumors) and patient tumors harboring the selected alteration, or the selected subtype (ST tumors). This results in four tumor groups for downstream analysis including the following: WT primary tumors, ST primary tumors, WT metastatic tumors, and ST metastatic tumors. After defining groups, the algorithm proceeds in three sequential steps: 1) genomic analysis, 2) transcriptomic analysis, and 3) clinical analysis.

For genomic analysis, primary and metastatic alteration data were used to calculate alteration frequencies for Gains and Losses. Fisher’s exact test was used to compute the statistical enrichment of alterations between the four tumor groups. Alterations present at a frequency of 2% or greater in ST primary tumors compared to WT primary tumors were captured and defined as primary tumor subtype co-alterations. Primary tumor subtype co-alterations that were present at a frequency of 2% or greater in ST metastatic tumors compared to ST primary tumors were selected for further analysis. Hits from the genomic analysis can be filtered above the 2% baseline by adjusting the primary co-alteration frequency difference and metastatic co-alteration frequency difference filtering parameters in the ProstaMine application.

For transcriptomic analysis, differential gene expression was computed by comparing the mean expression of primary ST tumors with and without the alteration and metastatic ST tumors with and without the alteration. Gains with a negative fold change in gene expression and Losses with a positive fold change in gene expression in primary and metastatic tumors were removed from the analysis. Statistical significance for the difference in gene expression was computed using a Student’s t-test. Hits from the transcriptomic analysis can be filtered by adjusting the concordant DGE FDR filtering parameter in the ProstaMine application.

For clinical data analysis, ProstaMine computed the association of each alteration’s gene expression with the Gleason grade group and progression-free survival. For each alteration, ST and WT primary tumors were median-stratified by gene expression into upper and lower groups. A Fisher’s exact test was used to compute the enrichment of the Gleason grade group ≥ 8 in the upper *versus* lower groups. The logrank test was used to compute statistical differences in progression-free survival times between these same groups. Alterations lacking concordant Gleason grade group enrichment and progression-free survival differences were removed. Alterations lacking concordant metastasis and progression-free survival associations were also removed. Alterations with a survival difference of *p* ≤ 0.2 in ST tumors and *p* ≥ 0.3 in WT tumors were captured as hits. Hits from the clinical analysis can be filtered below the *p* = 0.2 baseline by adjusting the survival *p*-value filtering parameter in the ProstaMine application.

We developed a prioritization scheme for ProstaMine hits. The effect size for each hit in primary co-alteration, metastasis, and progression-free survival was ranked and then normalized by the total number of hits (Eq. 1). The final score of subtype-specific aggressiveness was calculated by weighting the normalized rank for the co-alteration frequency difference in ST primary tumors, co-alteration frequency difference in ST metastatic tumors, and association with progression-free survival (Eq. 2):

$$Normalized\;Rank=1-\;\left(Rank\;Order/Total\;Number\;of\;Hits\right),$$ (1)

ProstaMine ScoreHit=0.3·Normalized RankPrimary Alteration frequency+0.3·Normalized RankMetastatic Alteration frequency+0.4·Normalized RankSurvival p−val, (2)

### 2.7 Analysis of NKX3-1-loss and RB1-loss prostate cancer

For *NKX3-1-*loss and *RB1*-loss prostate cancer, we set ProstaMine filtering parameters as follows: primary and metastatic Co–alteration rate difference = 0.05, primary and metastatic co-alteration FDR = 0.05, primary and metastatic DGE FDR = 0.2, and survival p-val = 0.05. For enrichment analysis in Metascape, we used the default settings for the Metascape’s Express Analysis option (Zhou et al., 2019).

### 2.8 Data and code availability

All genomic, transcriptomic, and clinicopathologic data used in this study are accessible through the *curatedPCaData* R package (Laajala et al., 2023). The corresponding code generated for performing all of the analyses in this study, creating ProstaMine and the Shiny application, is available at: github. com/MikeOrman/ProstaMine-Publication.git. ProstaMine is made available at: https://bioinformatics.cuanschutz.edu/prostamine.

## 3 Results

### 3.1 Alteration frequencies in primary prostate cancer reveal high-confidence alteration hotspots

**FIGURE 1:**
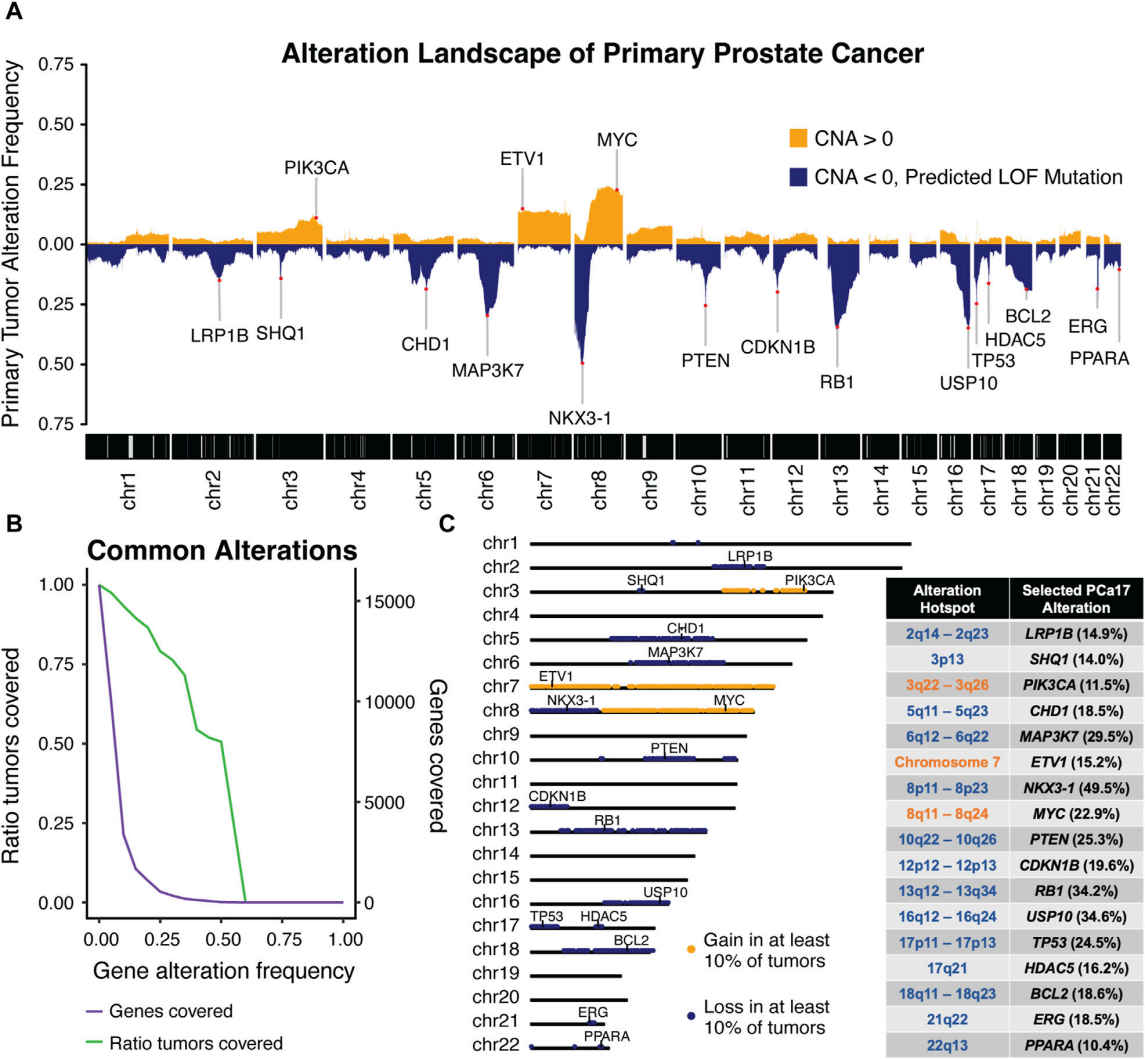
Integrated analysis of five independent molecular profiling studies in primary prostate was performed to generate **(A)** a combined alteration landscape of primary prostate cancer tumors and determine **(B)** primary tumor coverage over a range of alteration frequency cut-offs. An alteration frequency cut-off of 10% captured **(C)** 17 characteristic regions of alteration with each region harboring a canonical cancer-associated gene used to define the prostate cancer-17, or PCa17.

We first verified that the molecular data published across independent molecular profiling studies consistently identified *bona fide* PCa gene alterations using the Taylor et al. (2010), Baca et al. (2013), Barbieri et al. (2012), Hieronymus et al. (2014), and TCGA cohorts (Supplementary Figure S1) (Taylor et al., 2010; Barbieri et al., 2012; Baca et al., 2013; Hieronymus et al., 2014; Abeshouse et al., 2015). Alteration data from these five profiling studies were harmonized into a singular alteration matrix containing 15,869 genes across 921 primary PCa tumors. We calculated the somatic alteration frequency for each gene to define the PCa alteration landscape (Figure 1A).

The peaks observed in the genomic landscape span distinct genomic intervals (Figure 1A). To systematically identify these alteration hotspots, we computed the ratio of tumors covered by alterations meeting a given alteration frequency cut-off (Figure 1B). We observed a sharp decline in the number of genes captured at an alteration frequency cut-off of 10% and found that 93% of tumors were included at this cut-off. Using this threshold, we defined 17 alteration hotspots corresponding with the peaks depicted in the Figure 1A alteration landscape. Alteration hotspots captured 3,373 genes covering the following 17 chromosomal locations: 2q14-2q23, 3p13, 3q22-3q26, 5q11-5q23, 6q12-6q22, 7, 8p11-8p23, 8q11-8q24, 10q22-10q26, 12p12-12p13, 13q12-13q34, 16q12-16q24, 17p11-17p13, 17q21, 18q11-18q23, 21q22, and 22q13 (Figure 1C). These alteration hotspots were in agreement with previously reported regions of loss and gain measured in PCa tumors (Carter et al., 1990; Gao et al., 1995; Latil et al., 1997; Kibel et al., 1998; Ozen et al., 1998; Erbersdobler et al., 1999; Alers et al., 2000; Sattler et al., 2000; Dai et al., 2001; Verhagen et al., 2002; van Dekken et al., 2003; Paris et al., 2004; Ueda et al., 2005; Perner et al., 2006; Saramäki et al., 2006; Camp et al., 2007; Liu et al., 2007; Scheble et al., 2010; Kluth et al., 2018; 2015; Hieronymus et al., 2017). We found that each alteration hotspot harbored at least one cancer-associated gene that was present either directly at or very near the peak alteration frequency of the hotspot. We reasoned that grouping primary PCa tumors by these high-fidelity, cancer-associated alterations could be a powerful approach to stratify primary tumor aggressiveness in PCa. Thus, we selected a single cancer-associated gene to define each of the 17 alteration hotspots; we named this set of genes prostate cancer-17, or PCa17 (Figure 1C).

### 3.2 PCa17 alterations stratify primary prostate cancer tumors into four subgroups that associate with aggressive clinical features

**FIGURE 2:**
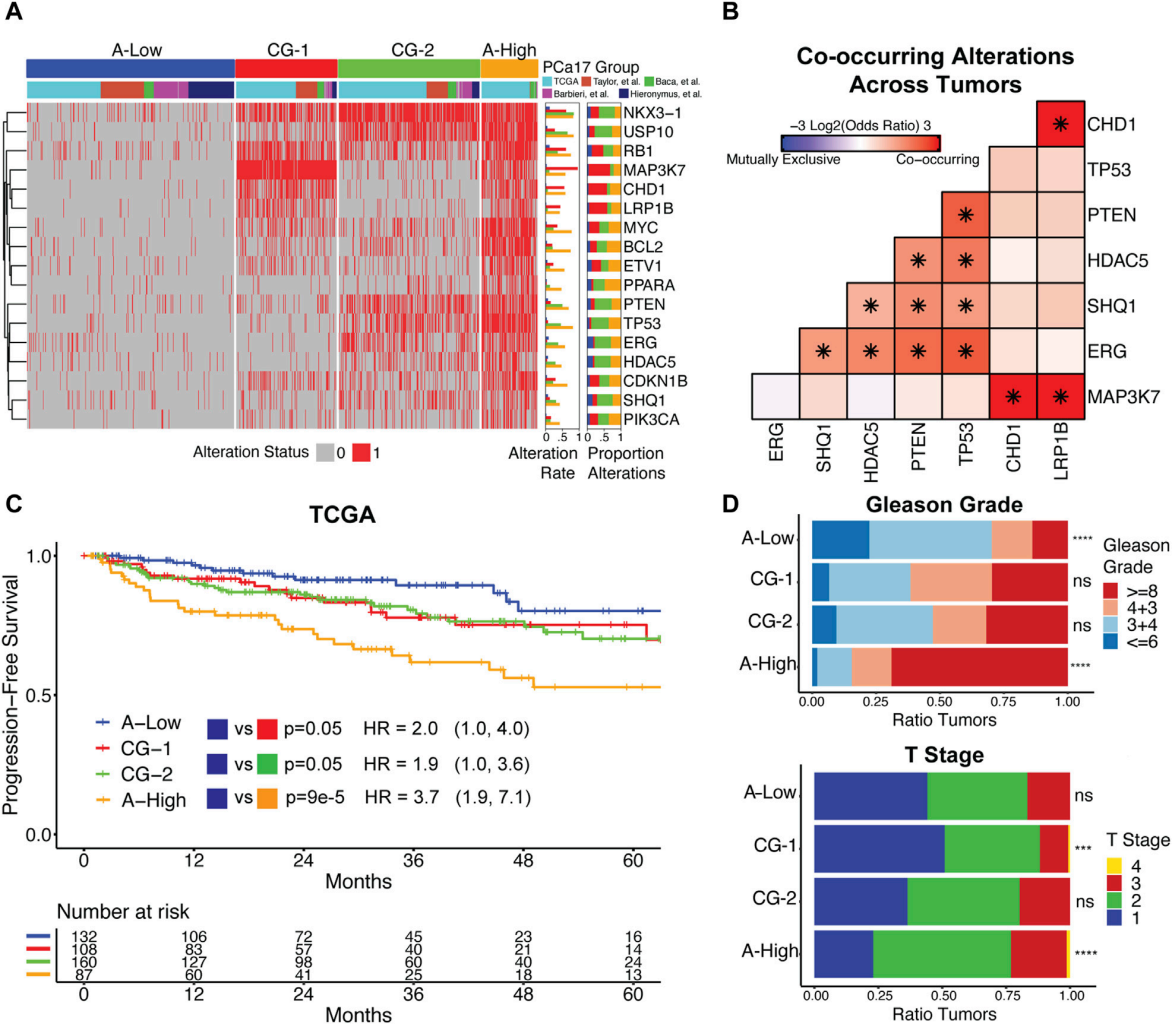
**(A)** Clustering primary prostate cancer tumors by PCa17 alterations defines four tumor subgroups with low overall alteration (A-Low), high overall alteration (A-High), and two distinct patterns of co-alteration (CG-1 and CG-2). **(B)** Mutual exclusivity analysis of *TP53, PTEN, ERG, HDAC5, SHQ1, MAP3K7, CHD1,* and *LRP1B* alterations across all primary tumors using Fisher’s exact test (*FDR<0.05). **(C)** Progression-free survival and proportional hazard ratios with 95% confidence interval comparing the tumor subgroups (A-High, CG-1, and CG-2) to A-Low in TCGA dataset. The logrank test was used to test for differences in progression-free survival times between groups. **(D)** Tumor grade and stage data for the four tumor subgroups. Fisher’s exact test was used to test for the enrichment of Gleason grade ≥8 and T2+ tumors for each tumor subgroup *versus* all other tumors (***FDR<0.001, ****FDR<0.0001).

We used the PCa17 alteration profiles and consensus clustering to define four distinct tumor subgroups (Figure 2A). One subgroup had a reduced number of overall alterations (A-Low, blue). Conversely, a second subgroup was characterized by a high number of alterations (A-High, orange) (Figure 2A). Certain alterations were enriched in one of the two remaining groups, co-alteration group 1 (CG-1, red) and co-alteration group 2 (CG-2, green) (Figure 2A). Alterations in *ERG, PTEN, SHQ1, TP53,* and *HDAC5* were depleted in CG-1 tumors at a frequency of 4%–13% and enriched in CG-2 tumors at a frequency of 48%–51%. Alterations in *MAP3K7*, *CHD1,* and *LRP1B* were enriched in CG-1 tumors at a frequency of 54%–63% and depleted in CG-2 tumors at a frequency of 9%–11% (Figure 2A). Given the patterns of enrichment and depletion observed in subgroups CG-1 and CG-2, we also tested for the co-alteration of *MAP3K7/CHD1/LRP1B* and *ERG/PTEN/SHQ1/TP53/HDAC5* across all primary tumors. We found that *MAP3K7*, *CHD1*, and *LRP1B* alterations formed a set of significantly co-occurring alterations (Figure 2B), while *ERG, TP53, HDAC5, PTEN,* and *SHQ1* alterations constituted a second set of significantly co-occurring alterations (Figure 2B). *NKX3-1* and *RB1* were the most commonly altered genes in A-Low tumors at a 12% frequency. In CG-1, CG-2, and A-high tumors, the *NKX3-1* alteration frequency increased to 61%–84%, and the *RB1* alteration frequency increased to 36%–75% (Figure 2A).

We analyzed associations between tumor subgroups and clinicopathologic features used in PCa staging. Survival analysis in TCGA dataset indicated that A-High tumors were the most likely to experience biochemical relapse over a 5-year period, followed by CG-1 and CG-2 tumors relative to A-Low tumors (Figure 2C). In the Taylor et al. dataset, we also found that CG-1 and CG-2 tumors were more likely to experience biochemical relapse over a 5-year period relative to A-Low tumors, although at modest significance (Supplementary Figure S3). Analysis of tumor grade and stage revealed that A-Low tumors were depleted of high-grade tumors (Gleason grade group ≥8), while A-High tumors were enriched with high-grade and T2+ tumors. CG-1 and CG-2 tumors displayed intermediate levels of tumor grade (Figure 2D). Clinical data on lymphatic spread (N1) showed enrichment in A-High tumors (Supplementary Figure S4). We also assessed the genomic instability of each group by the total number of CNAs. A-Low tumors had the least amount of CNAs relative to the other subgroups, while A-High tumors harbored the most CNAs (Supplementary Figure S4). Overall, these results show that grouping primary prostate cancer tumors by PCa17 alterations can stratify them into groups of less aggressive (A-Low) to intermediate (CG-1 and CG-2) to more aggressive characteristics (A-High).

### 3.3 ProstaMine: a computational tool for data mining subtype-specific co-alterations associated with PCa aggressiveness

**FIGURE 3:**
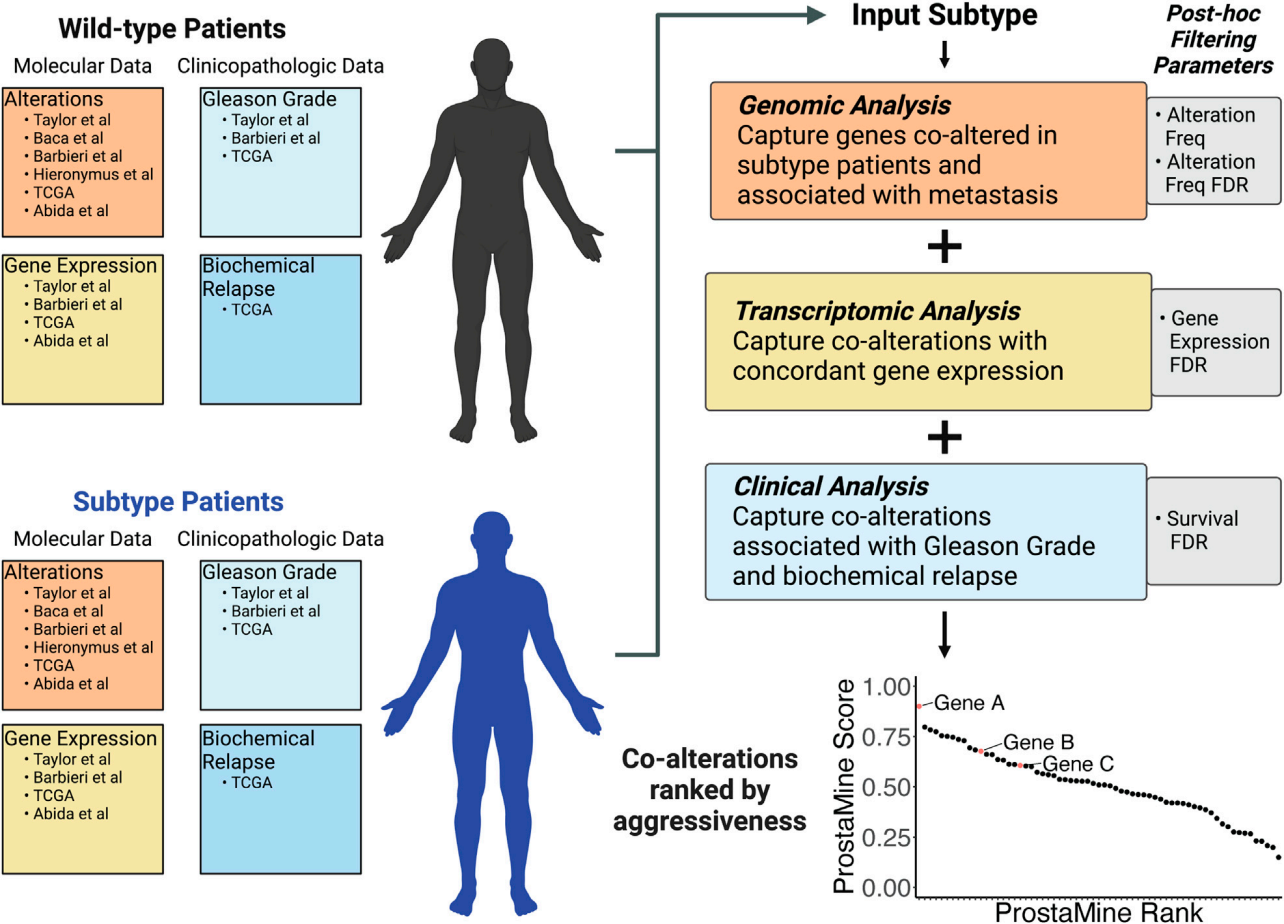
ProstaMine is a bioinformatics tool that integrates molecular and clinical data across six independent molecular profiling studies to determine a ranked list of subtype-specific co-alterations associated with aggressive features in prostate cancer.

Analysis of primary tumors confirmed many high-abundance molecularly defined subtypes in PCa. Considering the high abundance of these subtypes in primary PCa tumors, our goal was to define additional co-occurring alterations that may cooperate with these common single-genomic alterations to promote aggressive disease. We developed ProstaMine to be a publicly accessible bioinformatics tool that prioritizes subtype-specific co-alterations associated with metastasis and biochemical relapse in PCa. ProstaMine integrates tumor data on somatic CNAs, single-base substitutions, gene expression, biochemical relapse, and Gleason grade group across six independent PCa cohorts. ProstaMine leverages these data to identify co-alterations associated with aggressive tumors from molecularly defined Loss and Gain subtypes (Figure 3).

ProstaMine has the capability to mine any user-selected molecular subtype. Molecular alterations occurring early in tumor evolution are particularly good for ProstaMine because these subtypes are likely to contain many co-alterations that push the subtype into an aggressive state over the course of tumor development and progression. Recent work has shown that *NKX3-1* and *RB1* copy number losses are among the first alterations to occur in the evolution of prostate cancer (Espiritu et al., 2018). We reasoned that there are co-alterations that coordinate with *NKX3-1* and *RB1* losses to drive tumor progression, and we can use ProstaMine to find these co-alterations.

#### 3.3.1 Co-alterations associated with aggressiveness in NKX3-1-loss prostate cancer

**FIGURE 4:**
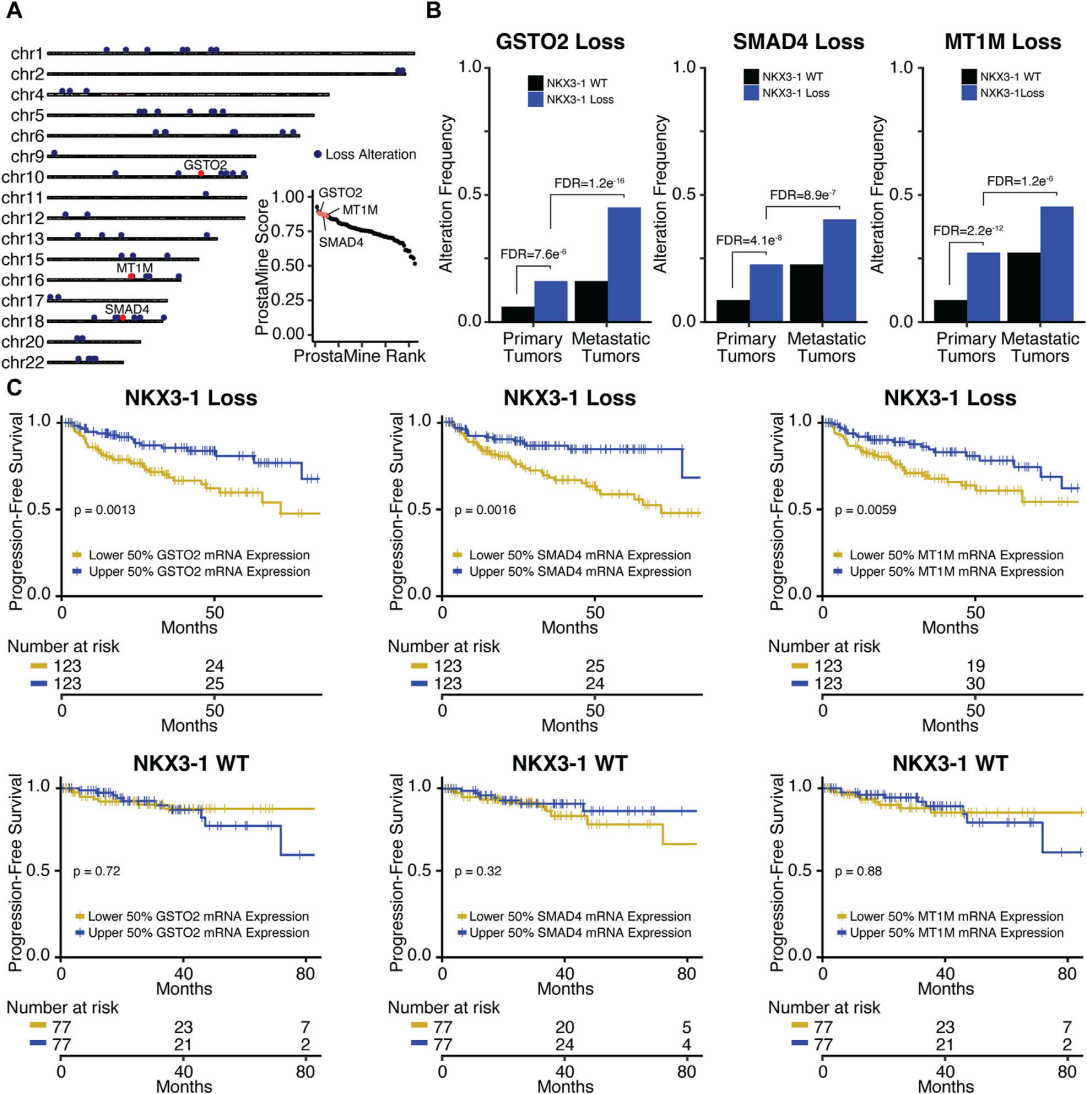
Application of ProstaMine to *NKX3-1-*loss prostate cancer **(A)** identified 73 loss co-alterations associated with aggressiveness with *GSTO2*, *SMAD4*, and *MT1M* loss alterations being top hits. Association of *GSTO2, SMAD4,* and *MT1M* hits with **(B)** *NKX3-1-*loss and metastasis and **(C)** progression-free survival. Fisher’s exact test was used to test for differences in alteration enrichment in **(B)** and the logrank test was used to test for differences in progression-free survival times in **(C)**.

In *NKX3-1-*loss tumors, ProstaMine identified 73 Loss co-alterations distributed across 16 chromosomal locations: 1p13-1p34, 2q37, 4p15-4p16, 5q12-5q23, 6q13-6q27, 9p24, 10q11-10q26, 11q22, 12p11-12p13, 13q12-13q33, 15q21-15q25, 16q13-16q24, 17p13, 18q12-18q23, 20p11, and 22q11-22q12 (Figure 4A). The third ranked single gene hit identified by ProstaMine was *GSTO2*, followed by *SMAD4* and *MT1M*, which were ranked sixth and eighth, respectively. *GSTO2* and *MT1M* have been described as tumor suppressors in cancer, and *SMAD4* is listed as a tumor suppressor gene in the COSMIC Cancer Gene Census (Mao et al., 2012; Sondka et al., 2018; Terayama et al., 2020; Xu et al., 2020; Li et al., 2021, 2023; Sumiya et al., 2022)*.* GSTO2 and MT1M functions have not been reported as factors in PCa progression, whereas loss of SMAD4 function has been shown to drive tumor growth and metastasis (Ding et al., 2011). Losses of *GSTO2*, *SMAD4*, and *MT1M* were significantly enriched in *NKX3-1-*loss primary tumors at a frequency of 10%–19% above primary WT tumors (Figure 4B). These alterations were enriched in metastatic *NKX3-1-*loss tumors at a frequency of 18%–29% above primary *NKX3-1-*loss tumors (Figure 4B). *GSTO2*, *SMAD4*, and *MT1M* co-alterations also had concordant and significantly reduced gene expression in primary and metastatic *NKX3-1-*loss tumors (Supplementary Figure S5B). Reduced gene expression of *GSTO2, SMAD4,* and *MT1M* was significantly associated with shorter time to biochemical relapse in *NKX*3-1-loss tumors but not in *NKX3-1-*WT tumors (Figure 4C). Reduced gene expression of *GSTO2, SMAD4,* and *MT1M* was also significantly associated with high-grade tumor histology (Gleason grade group ≥ 8) in *NKX3-1-*loss tumors (Supplementary Figure S5C).

*GSTO2* encodes a glutathione transferase involved in cellular detoxification. GSTO2 functions as a tumor suppressor through p38-mediated MAPK signaling in esophageal and squamous skin cell carcinoma (Terayama et al., 2020; Sumiya et al., 2022)*. SMAD4* encodes a transcription factor serving as the central regulator of the TGFb-activated and BMP4-activated SMAD signaling pathways. SMAD4 provides a barrier to metastatic progression in *PTEN*-null mouse prostates, and when deleted, it drives highly aggressive prostate cancer that metastasizes to the lymph node and lung (Ding et al., 2011). *MT1M* encodes a metallothionein protein that functions as a tumor suppressor by downregulating the NF-kB pathway activity and subsequent proliferation in hepatocellular carcinoma (Mao et al., 2012). In lung carcinoma, MT1M overexpression inhibits cell viability and migration through MDM2/p53 signaling, and in esophageal carcinoma, it inhibits the epithelial–mesenchymal transition (EMT) through the SOD1/PI3K signaling axis (Xu et al., 2020; Li et al., 2021). Taken together, the tumor suppressive mechanisms of GSTO2 and MT1M in these contexts suggest that p38, NF-kB, MDM2/p53, and SOD1/PI3K signaling may be contributing to aggressiveness in *NKX3-1*-loss tumors.

Pathway analysis of ProstaMine hits for *NKX3-1*-loss tumors identified several enriched signatures that are related to *fatty acid metabolism, metabolism of lipids,* and *autophagy* (Supplementary Figure S5A) (Zhou et al., 2019). In prostate cancer, altered fatty acid metabolism provides additional substrates for growth and signaling molecules that promote cancer cell proliferation, invasion, metastasis, and immune evasion. These processes are mediated by a number of molecular players including AR, PTEN/PI3K/AKT, c-Myc, and AMPK (Sena and Denmeade, 2021). In healthy tissues, autophagy controls the recycling of cellular material to maintain homeostasis; however, in PCa, the role of autophagy is contextual, and it can have both tumor suppressive and promotional effects (Loizzo et al., 2022). Taken together, pathway analysis of ProstaMine hits in *NKX3-1*-loss tumors suggests that dysregulated fatty acid metabolism and autophagy are important processes contributing to aggressiveness in *NKX3-1*-loss PCa.

#### 3.3.2 Co-alterations associated with aggressiveness in RB1-loss prostate cancer

**FIGURE 5:**
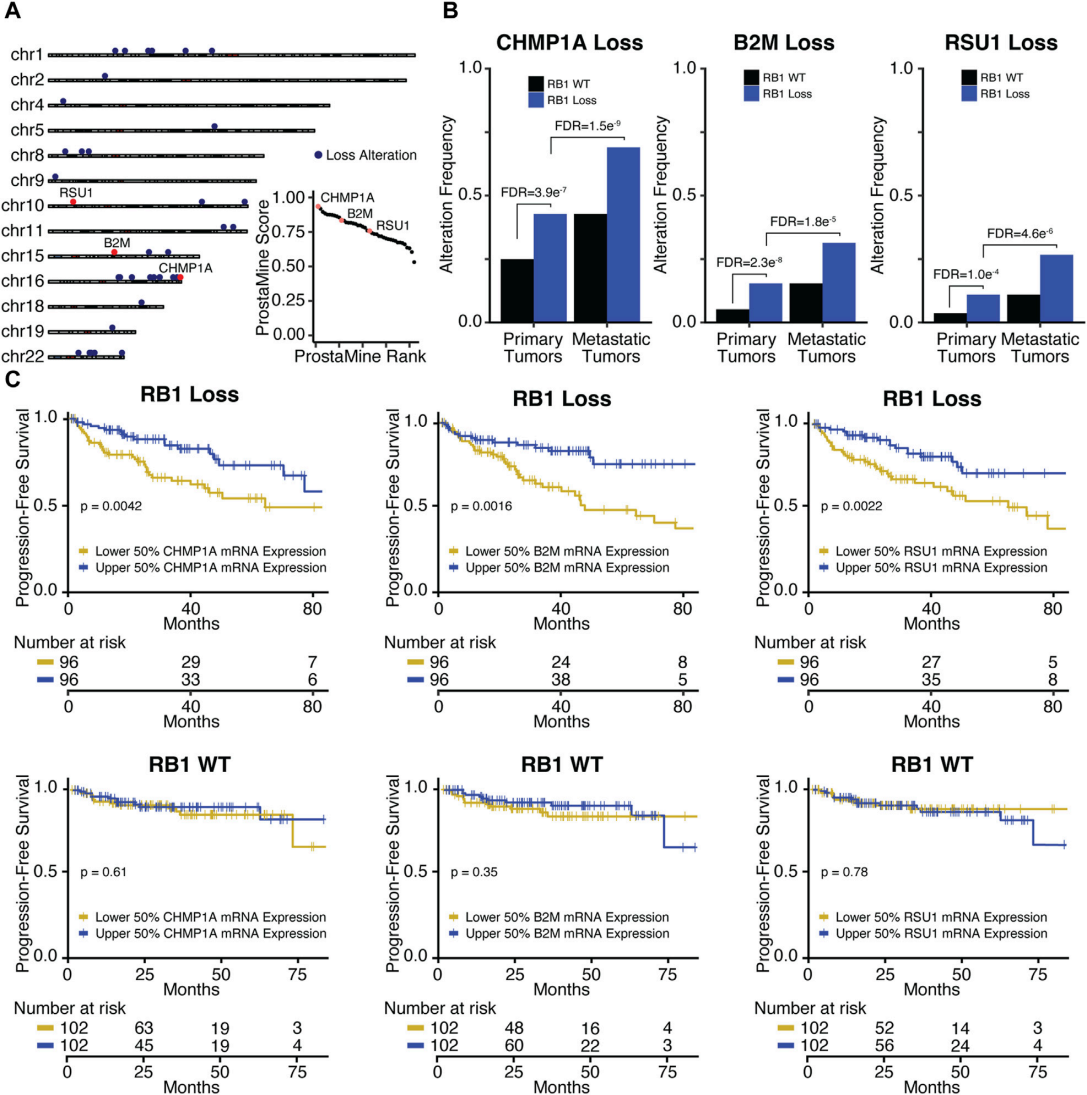
Application of ProstaMine to *RB1-*loss prostate cancer **(A)** identified 42 loss co-alterations associated with aggressiveness with *CHMP1A, B2M,* and *RSU1* loss alterations being top hits. Association of *CHMP1A, B2M,* and *RSU1* hits with **(B)** *RB1-*loss and metastasis, and **(C)** progression-free survival. Fisher’s exact test was used to test for differences in alteration enrichment in **(B),** and the logrank test was used to test for differences in progression-free survival times in **(C)**.

In *RB1-*loss tumors, ProstaMine identified 42 co-alterations distributed across 14 chromosomal locations: 1p13-1p34, 2p22, 4p16, 5q22, 8p21-8p23, 9p24, 10p13, 10q25-10q26, 11q23-11q24, 15q21-15q25, 16q11-16q24, 18q21, 19q13, and 22q11-22q12 (Figure 5A). The top ranked hit was *CHMP1A*, followed by *B2M* and *RSU1* which were ranked 11th and 23rd, respectively (Figure 5A). B2M, CHMP1A, and RSU1 functions have not been linked to PCa progression. These genes have all been reported to be tumor suppressors (Li et al., 2009; 2008; Gkretsi et al., 2019; Louca et al., 2019; Wang et al., 2021)*. CHMP1A, B2M,* and *RSU1* loss alterations were significantly enriched in *RB1*-loss primary tumors at a frequency of 7%–18% above *RB1*-WT primary tumors and were significantly enriched in *RB1-*loss metastatic tumors at a frequency of 16%–23% above *RB1-*loss primary tumors (Figure 5B). *CHMP1A* and *RSU1* loss alterations had significantly reduced concordant gene expression in primary and metastatic *RB1*-loss tumors (Supplementary Figure S6B). *B2M* loss alterations also showed a concordant reduction in gene expression but at moderate significance (Supplementary Figure S6B). Reduced gene expression of *CHMP1A, B2M,* and *RSU1* was significantly associated with decreased time to biochemical relapse in *RB1-*loss tumors but not in *RB1-*WT tumors (Figure 5C). Low *B2M* gene expression was significantly associated with high-grade tumor histology in *RB1-*loss tumors (Supplementary Figure S6C). *RB1-*loss tumors with low *CHMP1A* and *RSU1* gene expression also had more high-risk Gleason scores compared to those with high expression, although not statistically significant (Supplementary Figure S6C).

*CHMP1A* encodes a chromatin remodeling protein that functions as a tumor suppressor gene in pancreatic cancer cells through the activation of p53 and retinoic acid signaling (Li et al., 2008; Li et al., 2009)*. B2M* encodes a component of the MHC class I antigen presentation machinery and functions in immune surveillance. Alteration of *B2M* is common across different cancer types, and evidence suggests that *B2M* loss drives poor response to immunotherapy through disruption of MHC class I protein expression at the cell surface (Wang et al., 2021). *RSU1* encodes a focal adhesion protein that suppresses v-Ras-dependent oncogenic transformation (Cutler et al., 1992) and has recently been shown that suppression of RSU1 increases cell invasion through increased MMP13 expression and STAT6 phosphorylation (Louca et al., 2019).

We observed an overlap in several ProstaMine hits between *RB1*-loss and *NKX3-1-*loss tumors. Most notably, *GSTO2* and *MT1M* alterations were also among the top hits in *RB1-*loss tumors and pathway analysis of *RB1*-loss hits *via* Metascape once again identified autophagy among the significantly enriched pathways. Identification of *GSTO2*- and *MT1M*-loss alterations by ProstaMine in *NKX3-1-*loss and *RB1-*loss contexts suggests these co-alterations may be general regulators of aggressiveness in PCa. Five hits were found to regulate autophagy in *NKX3-1*-loss and *RB1-*loss tumors with three of these five hits overlapping between the two tumor subtypes. These findings provide evidence for dysregulated autophagy as a factor involved in the aggressiveness of *RB1*-loss PCa and further suggest a role for dysregulated autophagy as a general contributor to PCa aggressiveness.

## 4 Discussion

By combining five independent molecular profiling studies in primary PCa, we systematically identified high-confidence alteration hotspots across the primary PCa genome and found associations with clinicopathologic features related to aggressiveness. The majority of genes present in the PCa17 alteration signature have well-characterized tumor-suppressive or oncogenic roles that drive PCa. Grouping patients by these PCa17 alterations revealed a positive relationship between total alterations and aggressive features in primary PCa and is consistent with prior work, showing that a high copy number alteration burden predicts prostate cancer relapse (Hieronymus et al., 2014). We showed the most commonly altered genes in A-Low tumors are *NKX3-1* and *RB1* and that their alteration frequencies increase in CG-1, CG-2, and A-High tumors. Thus, the loss of *NKX3-1* and *RB1* are found across the entire spectrum of disease aggressiveness and is consistent with their role as initiating alterations in the evolution of PCa (Espiritu et al., 2018).

Analysis of molecular and clinical progression in PCa has revealed two distinct evolutionary trajectories including *SPOP* mutation *→ CHD1* loss and *ERG* fusion *→ PTEN* loss (Liu et al., 2021). We confirmed the co-alteration between *ERG/PTEN* and further captured *SHQ1, HDAC5,* and *TP53* loss alterations as significantly co-altered with *ERG* and *PTEN*. These findings suggest *SHQ1, HDAC5,* and *TP53* loss alterations may also be molecular features that help promote progression in primary PCa. Although *SPOP* was not included in the PCa17 alteration signature due to an alteration frequency below the 10% cutoff, we still found that *CHD1*- and *MAP3K7*-loss alterations were significantly co-occurring, as reported previously (Rodrigues et al., 2015). Interestingly, *LRP1B* loss was tightly associated with *CHD1* and *MAP3K7* loss, suggesting that *LRP1B* loss alterations may have functional significance in *MAP3K7*-loss and *CHD1*-loss PCa.

The co-alterations identified by ProstaMine may also inform potential therapeutic targeting strategies for specific PCa molecular subtypes. Co-alterations defined by Gains may be directly targetable for genes with corresponding pharmacological inhibitor(s). Alternatively, co-alterations defined by Losses will point toward subtype-specific pathway dysregulation, and the dysregulated pathways can potentially be therapeutically targeted. In *NKX3-1-*loss and *RB1-*loss PCa, ProstaMine identified exclusively Loss co-alterations, following filtering. *MT1M* loss was a top ProstaMine hit in both *NKX3-1-*loss and *RB1-*loss prostate cancer and has not been reported as a factor involved in PCa progression. *MT1M* suppression is known to promote cell growth and stemness properties in gastric cancer cell lines through increased GLI1 expression (Li et al., 2023). Interestingly, SHH-GLI1 pathway components often show enhanced expression in tumor *versus* normal prostatic epithelia, and suppressing GLI1 expression in primary prostate tumor cell cultures inhibits cell proliferation (Sanchez et al., 2004). Taken together, the ProstaMine results and results from other cancer types suggest that targeted inhibition of GLI1 or the SHH-GLI1 pathway may reduce the aggressiveness of *NKX3-1-*loss and *RB1-*loss PCa. Additionally, our pathway analysis of ProstaMine hits showed enrichment of fatty acid metabolism in *NKX3-1-*loss PCa and enrichment of autophagy in both *NKX3-1-*loss and *RB1-*loss PCa. Identification of positive regulators of fatty acid metabolism that are overexpressed in *NKX3-1-*loss prostate cancer may provide attractive targets for inhibiting the aggressiveness of this subtype. Likewise, identification of positive regulators of autophagy overexpressed in *NKX3-1-*loss and *RB1-*loss prostate cancer may be effective targets for slowing the aggressiveness of both subtypes.

The development of ProstaMine was possible through the integration of different data types including CNAs, single-base substitutions, gene expression, and clinicopathologic features. When integrating these data types from the available independent PCa studies, we noted four of the six studies contained matched genomic, transcriptomic, and clinical data. Inclusion of additional independent PCa profiling studies with these matched data types would improve the statistical power of ProstaMine, particularly for low-frequency subtypes. We also considered the representation of primary *versus* metastatic tumors used in ProstaMine. We obtained 919 primary tumors and 484 metastatic tumors, with 446 of these metastatic tumors sourced from Abida et al. (2019) and the remaining 38 coming from Taylor et al. (2010) (Taylor et al., 2010; Abida et al., 2019). Addition of more metastatic tumor data from independent profiling studies would balance the representation of metastatic tumors and improve the performance of ProstaMine. Our approach did not consider any treatment information for primary and metastatic tumor samples. Including treatment information would allow ProstaMine to interrogate the role of treatments in PCa subtypes.

The analysis of co-occurring alterations in cancer is a powerful approach for identifying subtype-specific mechanisms, driving disease development, progression, and metastasis. ProstaMine builds on this approach through integration of molecular and clinical data across multiple independent cancer profiling studies and is the first tool for the identification of molecular subtype-specific drivers of aggressive phenotypes in PCa.

## 5 Conclusion

We developed ProstaMine to identify co-alterations associated with metastasis and biochemical relapse in molecular subtypes of PCa. ProstaMine was applied to *NKX3-1*-loss and *RB1*-loss tumors and identified co-altered genes that function in canonical PCa signaling pathways including MAPK, NF-kB, p53, SMAD, and PI3K. These co-alterations also function in fatty acid metabolism and autophagy processes. ProstaMine is available to the larger research community to identify candidate genes and generate hypotheses on the mechanisms that drive aggressiveness in molecularly defined subtypes of PCa.

## Data Availability

The original contributions presented in the study are included in the article/Supplementary Material; further inquiries can be directed to the corresponding author.
