# Supplementary material for: ProstaMine: a bioinformatics tool for identifying subtype-specific co-alterations associated with aggressiveness in prostate cancer
Source: Front Pharmacol. 2024 May 1;15:1360352. doi: 10.3389/fphar.2024.1360352 (PMC11094266; doi:10.3389/fphar.2024.1360352)
Supplement: Supplementary file 1 [file DataSheet1.pdf]

Supplementary Material

ProstaMine: A bioinformatics tool for identifying subtype-specific co-alterations associated with aggressiveness in prostate cancer

Michael V. Orman, Varsha Sreekanth, Teemu D. Laajala, Scott D. Cramer, and James C. Costello*

*** Correspondence:**
James.Costello@cuanschutz.edu

Supplementary Figures

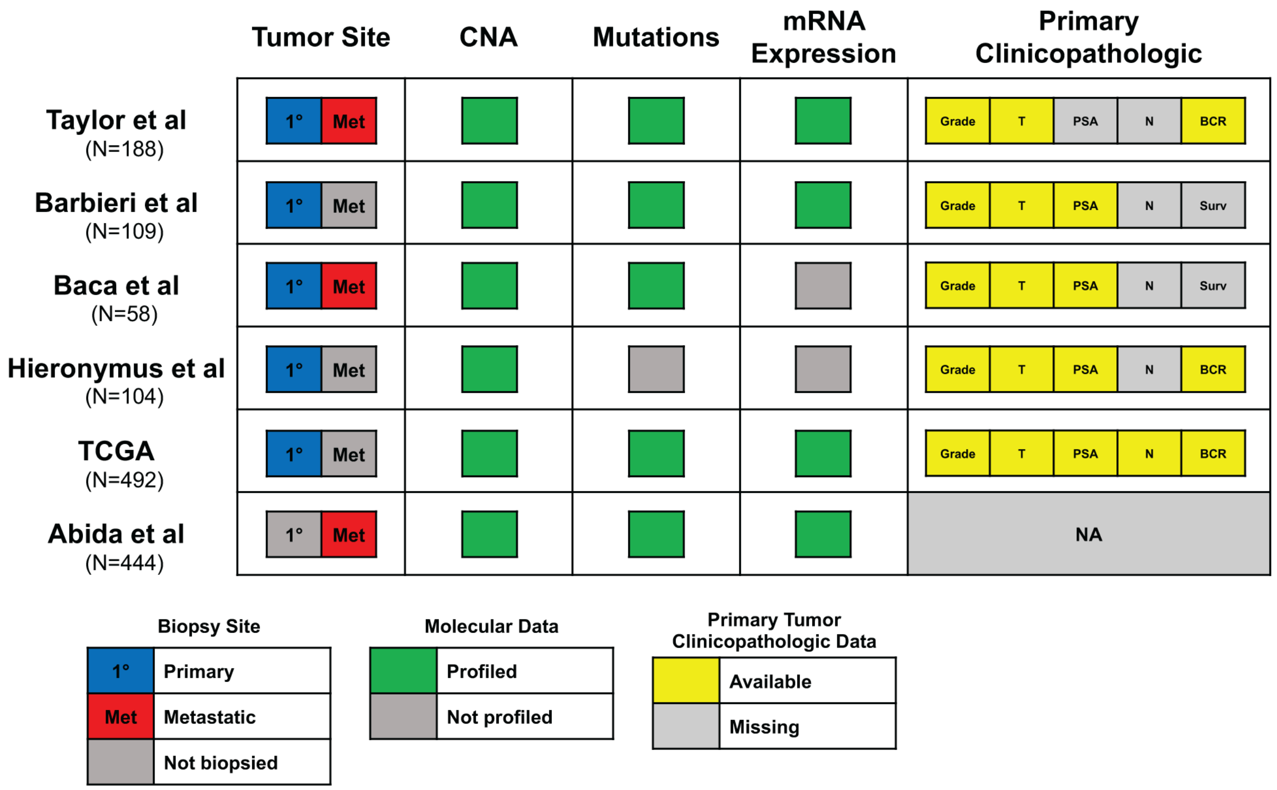

Supplementary Figure S1. Overview of different data types and corresponding data availability. Six molecular profiling studies in prostate cancer were sourced for conducting integrated analysis and creating the ProstaMine program.

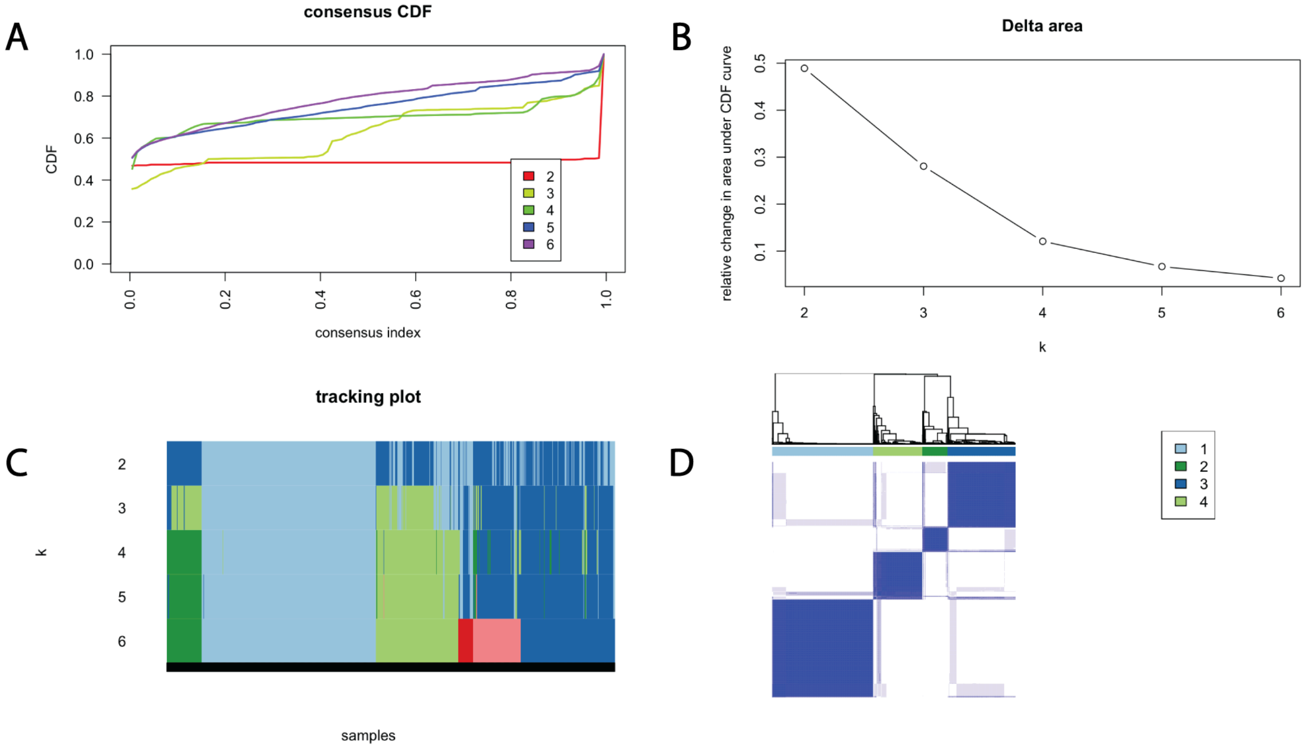

Supplementary Figure S2: Consensus clustering results for determining the number of primary prostate cancer tumor subgroups. Consensus clustering spanning k = 2 to k = 6 groups was tested to yield the resulting **(A)** CDF curve, **(B)** change in area under the CDF curve, and **(C)** tracking plot. **(D)** Consensus matrix for the selected k = 4 primary tumor subgroups.

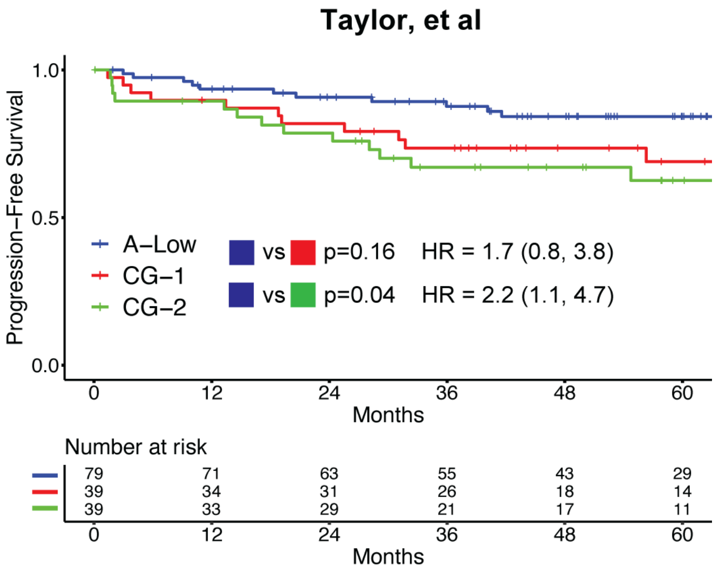

Supplementary Figure S3: Progression-free survival and proportional hazard ratios with 95% confidence interval comparing the tumor subgroups (CG-1 and CG-2) to A-Low in the Taylor, et al dataset. The A-High group was excluded from this analysis because only 1 sample was present in this group**.** The log-rank test was used to test for differences in survival times between groups.

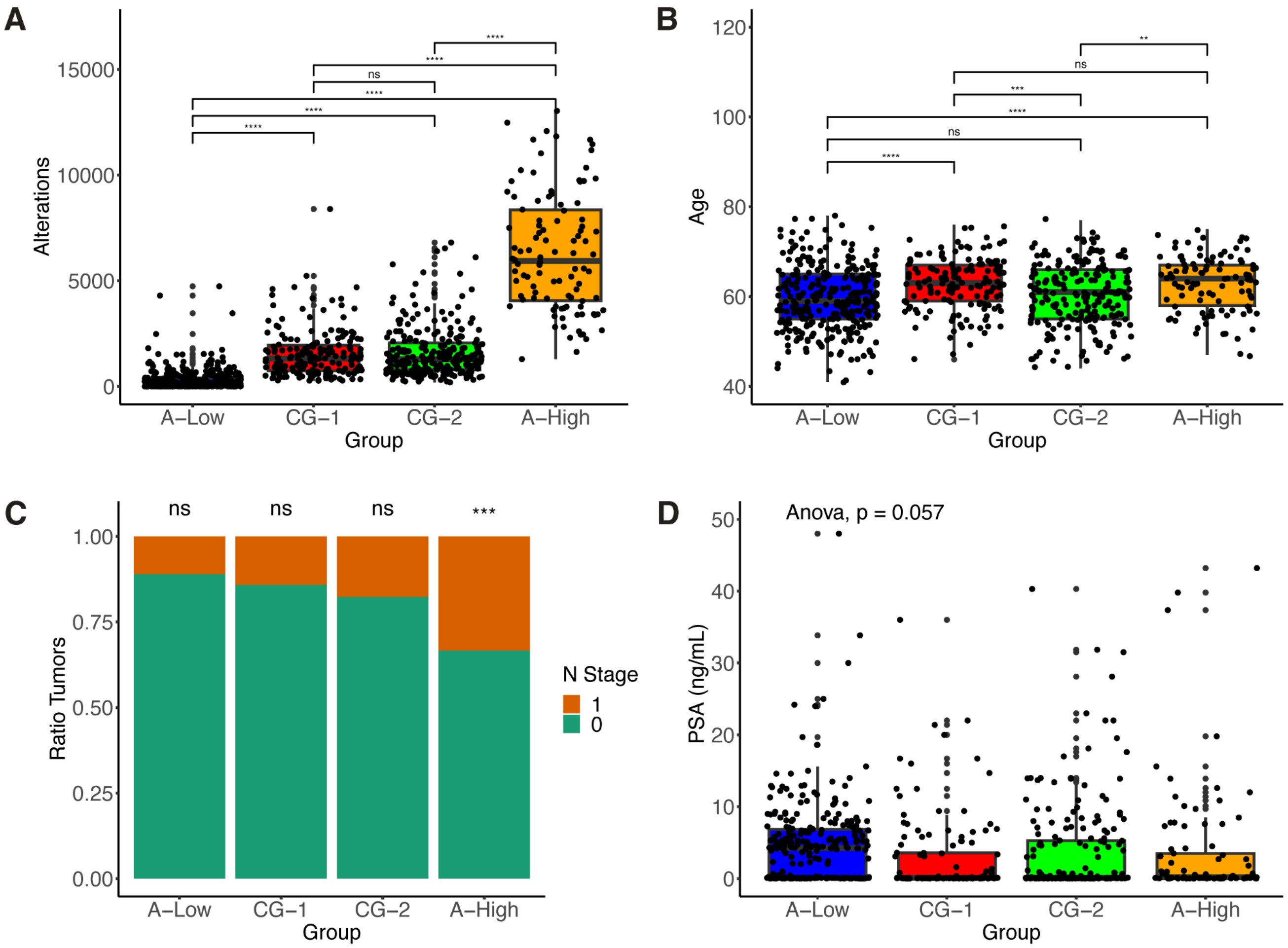

Supplementary Figure S4. Additional clinicopathologic features for each of the four primary prostate cancer tumor subgroups including **(A)** the total number of genes altered, **(B)** patient age, **(C)** N stage, and **(D)** prostate-specific antigen (PSA) level. Student's t-test was used to test for differences between tumor subgroups in **A** and **B**. ANOVA was used to test for differences between groups in **D**. Fisher's exact test was used to test for N1 enrichment in each tumor subgroup vs. all other tumors in **C.** (**FDR<0.01, ***FDR<0.001, ****FDR<0.0001).

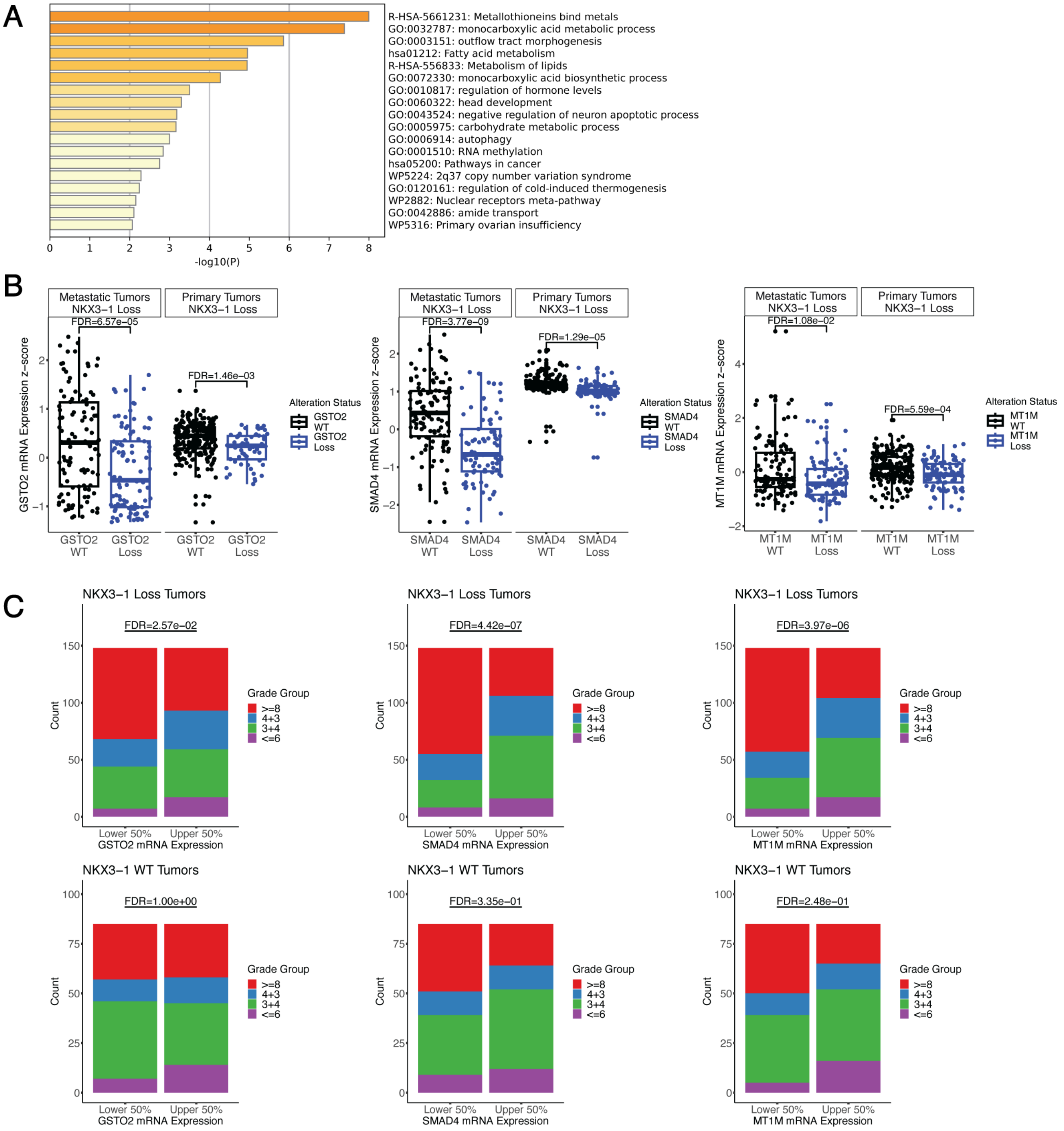

Supplementary Figure S5. Additional analysis of ProstaMine hits in *NKX3-1*-loss prostate cancer. **(A)** Pathway enrichment analysis via Metascape for the 73 loss co-alterations identified by ProstaMine. **(B)** Gene expression analysis of *GSTO2, SMAD4,* and *MT1M* loss alterations. **(C)** Gleason Grade Group analysis of *GSTO2, SMAD4,* and *MT1M* loss alterations. Student's t-test was used to test for gene expression differences in **B** and Fisher's exact test was used to test for enrichment of Gleason Grade ≥ 8 tumors in **C**.

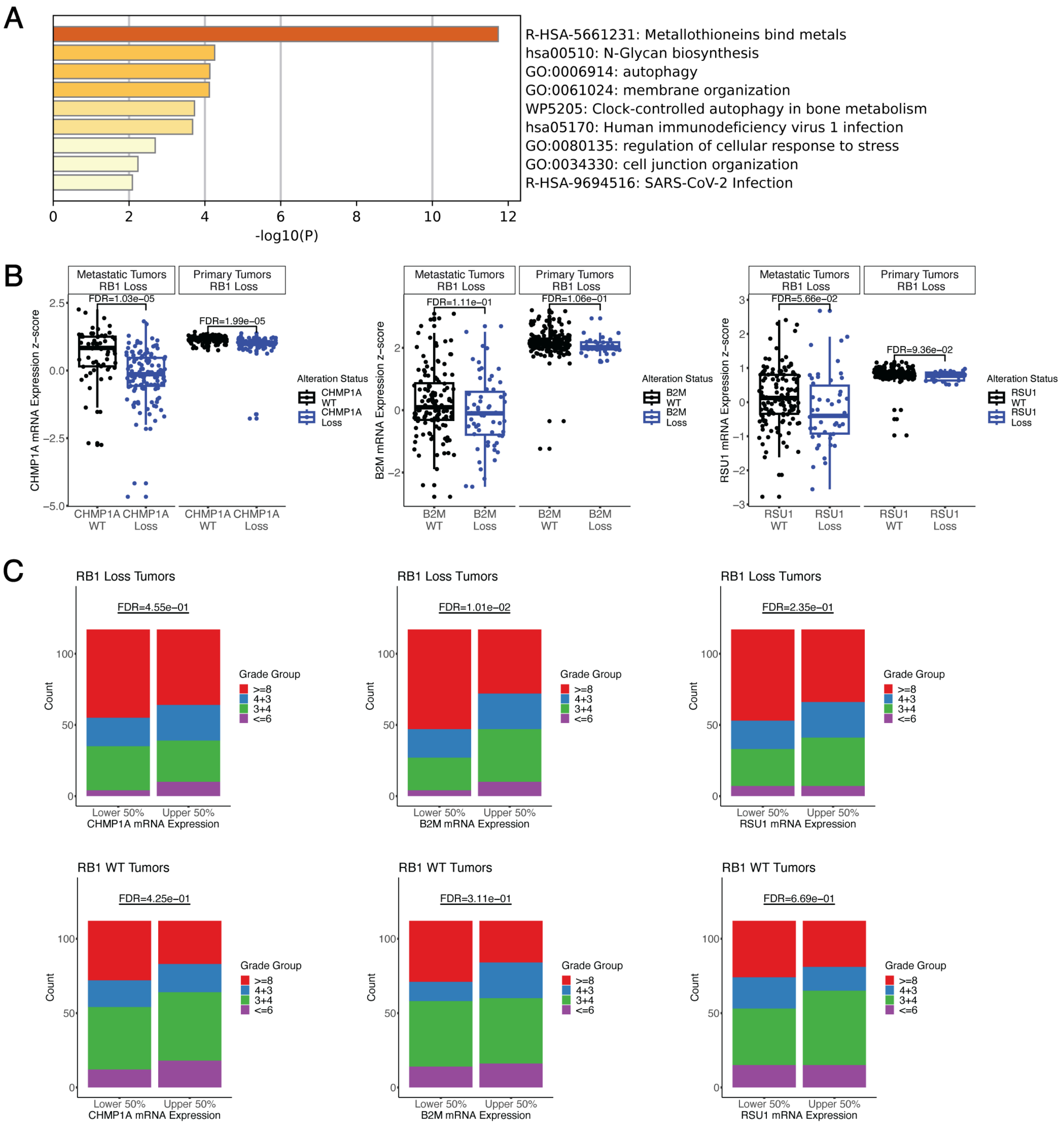

Supplementary Figure S6. Additional analysis of ProstaMine hits in *RB1*-loss prostate cancer. **(A)** Pathway enrichment analysis via Metascape for the 42 loss co-alterations identified by ProstaMine. **(B)** Gene expression analysis of *CHMP1A, B2M,* and *RSU1* loss alterations. **(C)** Gleason Grade Group analysis of *CHMP1A, B2M,* and *RSU1* loss alterations. Student's t-test was used to test for gene expression differences in **B** and Fisher's exact test was used to test for enrichment of Gleason Grade ≥ 8 tumors in **C**.
